# Minocycline Decreases Liver Injury after Hemorrhagic Shock and Resuscitation in Mice

**DOI:** 10.1155/2012/259512

**Published:** 2012-06-07

**Authors:** Christoph Czerny, Andaleb Kholmukhamedov, Tom P. Theruvath, Eduardo N. Maldonado, Venkat K. Ramshesh, Mark Lehnert, Ingo Marzi, Zhi Zhong, John J. Lemasters

**Affiliations:** ^1^Center for Cell Death, Injury & Regeneration, Department of Pharmaceutical & Biomedical Sciences, Medical University of South Carolina, Charleston, SC 29425, USA; ^2^Department of Trauma Surgery, J. W. Goethe University, 60590 Frankfurt am Main, Germany; ^3^Department of Biochemistry & Molecular Biology, Medical University of South Carolina, Charleston, SC 29425, USA

## Abstract

Patients that survive hemorrhage and resuscitation (H/R) may develop a systemic inflammatory response syndrome (SIRS) that leads to dysfunction of vital organs (multiple organ dysfunction syndrome, MODS). SIRS and MODS may involve mitochondrial dysfunction. Under pentobarbital anesthesia, C57BL6 mice were hemorrhaged to 30 mm Hg for 3 h and then resuscitated with shed blood plus half the volume of lactated Ringer's solution containing minocycline, tetracycline (both 10 mg/kg body weight) or vehicle. Serum alanine aminotransferase (ALT), necrosis, apoptosis and oxidative stress were assessed 6 h after resuscitation. Mitochondrial polarization was assessed by intravital microscopy. After H/R with vehicle or tetracycline, ALT increased to 4538 U/L and 3999 U/L, respectively, which minocycline decreased to 1763 U/L (*P* < 0.01). Necrosis and TUNEL also decreased from 24.5% and 17.7 cells/field, respectively, after vehicle to 8.3% and 8.7 cells/field after minocycline. Tetracycline failed to decrease necrosis (23.3%) but decreased apoptosis to 9 cells/field (*P* < 0.05). Minocycline and tetracycline also decreased caspase-3 activity in liver homogenates. Minocycline but not tetracycline decreased lipid peroxidation after resuscitation by 70% (*P* < 0.05). Intravital microscopy showed that minocycline preserved mitochondrial polarization after H/R (*P* < 0.05). In conclusion, minocycline decreases liver injury and oxidative stress after H/R by preventing mitochondrial dysfunction.

## 1. Introduction

Trauma and surgical procedures, including gastrointestinal and hepatobiliary surgery, can lead to severe hemorrhage and hypovolemic shock. Fluid resuscitation after less than one hour of severe hemorrhagic shock restores hemodynamics and typically leads to full recovery. By contrast although restoring hemodynamics, resuscitation after greater than an hour may lead instead to multiple organ dysfunction syndrome (MODS), which is associated with mortality of 30% [1]. Effective strategies to extend this golden hour for resuscitation are therefore needed to improve the treatment of hemorrhagic shock and decrease the incidence of MODS and its lethal consequence. Hemorrhage/resuscitation (H/R) is an example of ischemia/reperfusion (I/R) and hypoxia/reoxygenation injuries, for which mitochondrial dysfunction plays a major pathophysiological role [2–4]. Moreover, the liver with its crucial involvement in metabolism and homeostasis is among the most frequently affected organs after hemorrhage-induced hypotension in humans [5].

I/R injury leads to both necrotic cell death and apoptosis. A common pathway for hepatic apoptosis and necrosis after I/R is the mitochondrial permeability transition (MPT) [6]. Opening of permeability transition (PT) pores in the mitochondrial inner membrane causes the MPT with consequent mitochondrial depolarization and uncoupling of oxidative phosphorylation. ATP depletion after uncoupling produces necrotic cell killing, the main pathway of cell death after I/R, whereas cytochrome *c* release due to MPT-driven mitochondrial swelling induces caspase-dependent apoptosis. Previously, experimental strategies to inhibit the MPT after liver transplantation in rats improved survival and decreased mitochondrial dysfunction [7]. 

Minocycline is a semisynthetic tetracycline antibiotic, which is protective against neurodegenerative disease, trauma, and hypoxia/ischemia [8–12]. Mechanisms by which minocycline exerts neuroprotection include inhibition of apoptotic pathways, decreased mitochondrial release of proapoptotic factors like cytochrome *c*, and upregulation of antiapoptotic Bcl-2 and inhibitor of apoptosis proteins (IAPs) [13, 14]. In orthotopic rat liver transplantation, minocycline cytoprotection against storage/reperfusion injury is mediated by suppression of the MPT through inhibition of the mitochondrial calcium uniporter [7]. Here, we investigated whether resuscitation with minocycline also decreases liver injury after H/R.

## 2. Materials and Methods

### 2.1. Chemicals and Reagents

Minocycline, tetracycline, rhodamine 123, and other reagents were purchased from Sigma-Aldrich (St. Louis, MO).

### 2.2. Animals

Male C57BL/6J mice (8–10 wk of age, 23–27 g) were obtained from Jackson Laboratory (Bar Harbor, ME). Animal protocols were approved by the Institutional Animal Care and Use Committee of the Medical University of South Carolina.

### 2.3. Hemorrhagic Shock and Resuscitation

After an overnight fast, mice were anesthetized with pentobarbital sodium (80 mg/kg body weight). Under spontaneous breathing, both femoral arteries were exposed and cannulated with polyethylene-10 catheters (SIMS Portex). The catheters were flushed with normal saline containing heparin (100 IU/l) before insertion. One catheter was connected via a transducer to a pressure analyzer (Micro-Med; Louisville, KY), and blood was withdrawn via the second catheter into a heparinized syringe (10 units) over 5 min to a mean arterial pressure of 30 mm Hg. This pressure was maintained for 3 h by withdrawal or reinfusion of shed blood [15]. Body temperature was monitored and maintained at 37°C. After 3 h, mice were resuscitated with a syringe pump over 30 min with shed blood followed by a volume of lactated Ringer's solution corresponding to 50% of the shed blood volume [16, 17]. As indicated, the resuscitating Ringer's solution contained minocycline (10 mg/kg body weight), tetracycline (10 mg/kg), or vehicle. Doses of minocycline and tetracycline were based on a prior study [7]. Adequacy of resuscitation was determined by the restoration of blood pressure. Catheters were then removed, the vessels were ligated, and the groin incisions were closed. Sham-operated animals underwent the same surgical procedures, but hemorrhage was not carried out. No mortality in any group occurred over the course of the experiments.

For the determination of hemorrhage-/resuscitation-dependent liver damage, mice were anesthetized and killed by exsanguination 6 h after the end of resuscitation. For each mouse, the two right dorsal liver lobes were snap-frozen in liquid nitrogen. The remaining liver was flushed with normal saline, infused and fixed with 4% buffered paraformaldehyde through the portal vein, and embedded in paraffin sections.

### 2.4. Alanine Aminotransferase (ALT)

Blood samples to measure ALT were collected from the inferior vena cava 6 h after H/R for analysis by standard methods.

### 2.5. Histology

Sections (4 *μ*m) were stained with hematoxylin and eosin (H&E). Ten random fields were assessed for necrosis by standard morphologic criteria (e.g., loss of architecture, vacuolization, karyolysis, increased eosinophilia). Images were captured by an image analysis system (Olympus BH-2 Microscope; Micropublisher 5.0 RTV, Center Valley, PA), and the area percentage of necrosis was quantified using a computer program (BioQuant BQ Nova Prime 6.7, R&M Biometrics, Nashville, TN).

### 2.6. TUNEL

Terminal deoxynucleotidyl transferase-mediated dUTP nick-end labeling (TUNEL) was performed on paraffin sections using an *in situ* cell death detection kit (Roche Diagnostics, Penzberg, Germany). TUNEL-positive cells were counted by light microscopy in 10 random high-power fields (HPF). 

### 2.7. Caspase-3

Liver tissue (~100 mg) was homogenized (Polytron PT-MR2100, Kinematica, Luzern, Switzerland) in 1 mL of lysis buffer containing 0.1% 3[(3-cholamidopropyl)dimethylammonio]-propanesulfonic acid, 2 mM EDTA, 5 mM dithiothreitol, 1 mM Pefabloc, 10 ng/mL pepstatin A, 10 ng/mL aprotinin, 20 *μ*g/mL leupeptin, and 10 mM HEPES buffer, pH 7.4. The lysate was centrifuged at 15,000 rpm for 30 min. Activity of caspase-3 in the supernatant was determined using a Caspase-3 Colorimetric Assay Kit (R&D Systems, Minneapolis, MN) according to the manufacturer's instructions. Activity was normalized to protein concentration of each sample and expressed as fold increase compared to sham.

### 2.8. 4-Hydroxynonenal

Paraffin-embedded sections were deparaffinized, rehydrated, and incubated with polyclonal antibodies against 4-hydroxynonenal (4-HNE, Alpha Diagnostics; San Antonio, TX) in PBS (pH 7.4) containing 1% Tween 20 and 1% bovine serum albumin. Peroxidase-linked secondary antibody and diaminobenzidine (Peroxidase Envision Kit, DAKO) were used to detect specific binding. A Universal Imaging Metamorph image acquisition and analysis system (Chester, PA) incorporating an Axioskop 50 microscope (Carl Zeiss; Thornwood, NY) was used to capture and analyze the immunostained tissue sections at 40x magnification. The extent of labeling was determined in randomly selected fields as the percentage of area within a preset color range determined by the software. Data from each tissue section (10 fields/section) were pooled to determine means, as described previously [18].

### 2.9. Intravital Multiphoton Microscopy

At 4 h after H/R, mice were anesthetized with pentobarbital (50 mg/kg) and connected to a small animal ventilator via a tracheostomy and respiratory tube (22-gauge catheter), as described previously [19]. Laparotomy was performed, and a polyethylene-10 catheter was inserted into the distal part of the right colic vein. Using a syringe pump, rhodamine 123 (1 *μ*mol/mouse), a membrane potential-indicating fluorophore, was infused via the catheter over 10 min. After prone positioning of the mouse, the liver was gently withdrawn from the abdominal cavity and placed over a glass coverslip on the microscope stage. Rhodamine 123 fluorescence was excited with 820 nm light from a Chameleon Ultra Ti-Sapphire pulsed laser (Coherent, Santa Clara, CA) and imaged with a Zeiss LSM 510 NLO inverted laser scanning confocal microscope using a 63x 1.3 NA water-immersion objective lens. Green rhodamine 123 fluorescence was collected through a 525 ± 25 nm band pass filter. During image acquisition, the respirator was turned off for ~5 sec to eliminate movement artifacts from breathing. In 20 fields per liver, parenchymal cells were scored for bright punctate rhodamine 123 fluorescence representing hepatocytes with polarized mitochondria or dimmer diffuse cytosolic fluorescence representing hepatocytes with depolarized mitochondria. Image analysis was performed in a blinded fashion.

### 2.10. Statistical Analysis

Data are presented as means ± S.E., unless otherwise noted. Statistical analysis was performed by ANOVA plus Student-Newman-Keuls test, as appropriate, using *P* < 0.05 as the criterion of significance.

## 3. Results

### 3.1. Decreased ALT Release and Liver Necrosis after Resuscitation with Minocycline

**Figure 1:**
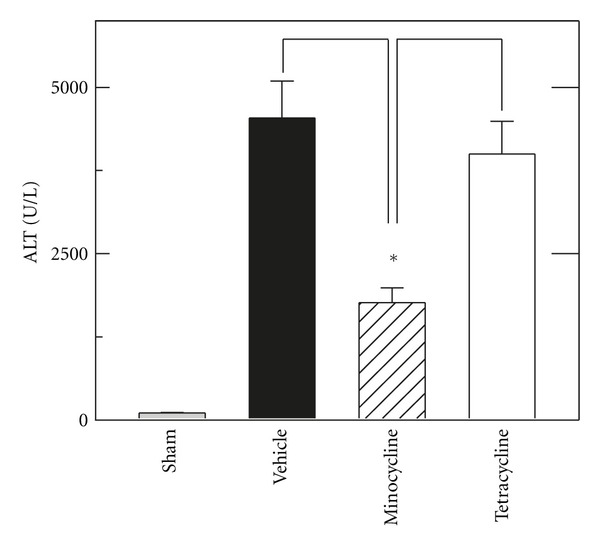
Minocycline decreases ALT release after hemorrhage and resuscitation. Mice were resuscitated with shed blood and then half the volume of lactated Ringer's solution containing tetracycline or minocycline (10 mg/kg body weight) or vehicle, as described in materials and methods. Serum ALT was assessed 6 h after resuscitation. Group sizes were sham, 4; vehicle, 7; minocycline, 7; tetracycline, 7. *P < 0.01 versus vehicle and tetracycline.

C57BL6 mice were hemorrhaged for 3 h and resuscitated with shed blood followed by half the volume of lactated Ringer solution, containing minocycline (10 mg/kg), tetracycline (10 mg/kg), or vehicle. As described previously [20], resuscitation restored mean arterial pressure to ~80 mm Hg, which was nearly identical to blood pressure before hemorrhage (data not shown). At 6 h postoperatively, sham-operated mice had serum ALT of  105 ± 15 U/L (Figure 1). After H/R, ALT after vehicle treatment increased to 4538 U/L ± 557 U/L, which decreased to  1763 ± 213 U/L after resuscitation with minocycline (*P* < 0.01). Identical treatment with tetracycline did not cause a statistically significant change of serum ALT (3999 ± 491 U/L) compared to vehicle (Figure 1).

**Figure 2:**
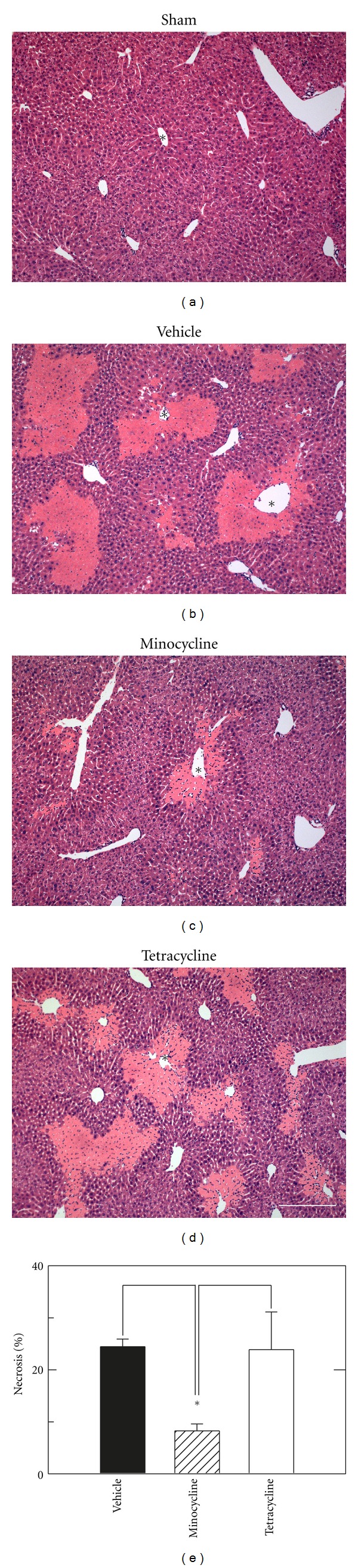
Minocycline decreases necrosis after hemorrhage and resuscitation. H/R was performed, as described in Figure 1. Necrosis was assessed by H&E histology at 6 h after sham operation (a) or resuscitation with vehicle, minocycline, or tetracycline (b–d). In (e), necrosis as percent area in liver sections was averaged from 5 livers per treatment group. Necrosis in sham-operated mice was absent and not plotted. *, central vein. Bar is 100 *μ*m. *P < 0.05 versus vehicle and tetracycline.

Liver injury was also assessed histologically at 6 h postoperatively. In sham-operated mice, liver histology was normal and indistinguishable from untreated mice (Figure 2(a) and data not shown). After H/R with vehicle and tetracycline treatments, large areas of necrosis developed 6 h postoperatively with a predominately pericentral and midzonal distribution, which was decreased after resuscitation with minocycline (Figures 2(b)–2(d)). Resuscitation with minocycline decreased hepatic necrosis from 24.5 ± 1.5% after vehicle to 8.3 ± 1.4% (*P* < 0.05) (Figure 2(e)). By contrast, resuscitation with tetracycline did not decrease liver necrosis after H/R (23.3 ± 1.5%) in comparison to vehicle treatment. Overall, minocycline treatment decreased hepatic necrosis by nearly two-thirds. 

### 3.2. Decreased Liver Apoptosis after Resuscitation with Minocycline and Tetracycline

**Figure 3:**
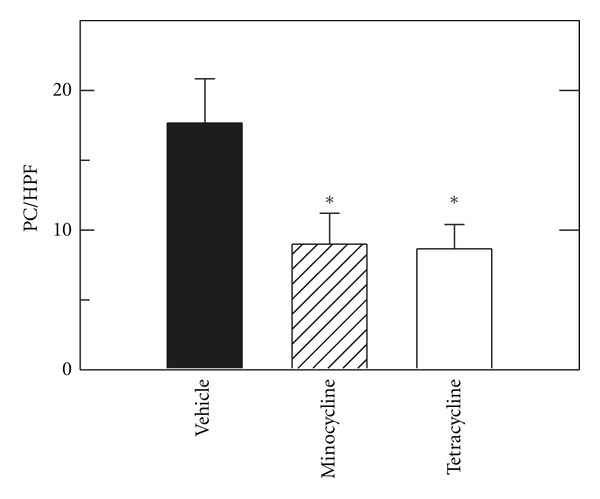
Minocycline and tetracycline decrease apoptosis after hemorrhage and resuscitation. H/R was performed, as described in Figure 1. Apoptosis of parenchymal cells was assessed by TUNEL in nonnecrotic areas at 6 h after sham operation or resuscitation with vehicle, minocycline, or tetracycline. The average number of TUNEL positive cells is plotted for each treatment group. TUNEL for sham was virtually zero and is not plotted. Bar is 50 *μ*m. *P < 0.05 versus vehicle.

TUNEL was performed on tissue sections to assess double-stranded DNA breaks that are characteristic of apoptosis. TUNEL-positive parenchymal cells were rare after sham operation, averaging less than one cell per high power field (HPF). At 6 h after H/R with vehicle, TUNEL of parenchymal cells in nonnecrotic areas increased to 17.7 ± 3.2 cells/HPF (Figure 3). Treatment with minocycline decreased TUNEL by half to 8.7 ± 1.7 cells/HPF (*P* < 0.05 compared to vehicle, Figure 3). After resuscitation with tetracycline, TUNEL-positive cells in nonnecrotic areas decreased to  9 ± 2.2 cells/HPF (*P* < 0.05 compared to vehicle, Figure 3) as well.

**Figure 4:**
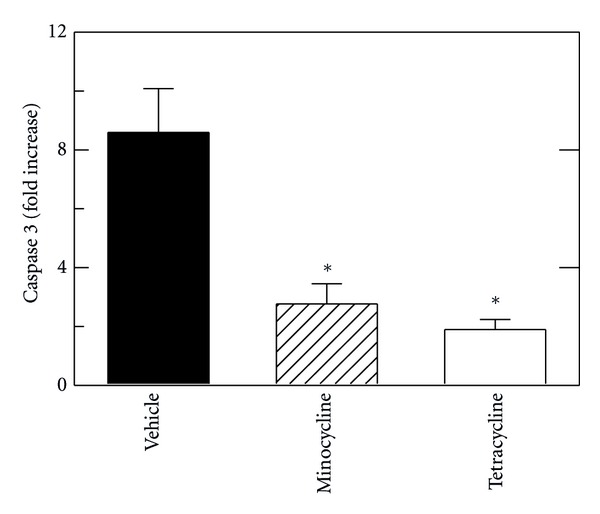
Minocycline and tetracycline decrease caspase 3 activation after hemorrhage and resuscitation. H/R was performed, as described in Figure 1, and caspase 3 activity was assessed in liver homogenates after sham operation or resuscitation with vehicle, minocycline, or tetracycline. Activity is expressed as fold increase over sham-operated mice. *P < 0.05 versus vehicle.

To further investigate the extent of apoptosis after minocycline and tetracycline treatment, caspase-3 activity was measured in liver extracts at 6 h after resuscitation with vehicle, minocycline and tetracycline in comparison to sham operation (Figure 4). After sham operation, caspase-3 activity was very low. After H/R with vehicle, caspase-3 activity increased 8.6-fold, which decreased to 2.8-fold after minocycline and to 2-fold after tetracycline (*P* < 0.05 compared to vehicle, Figure 4).

### 3.3. Decreased Oxidative Stress after Resuscitation with Minocycline

**Figure 5:**
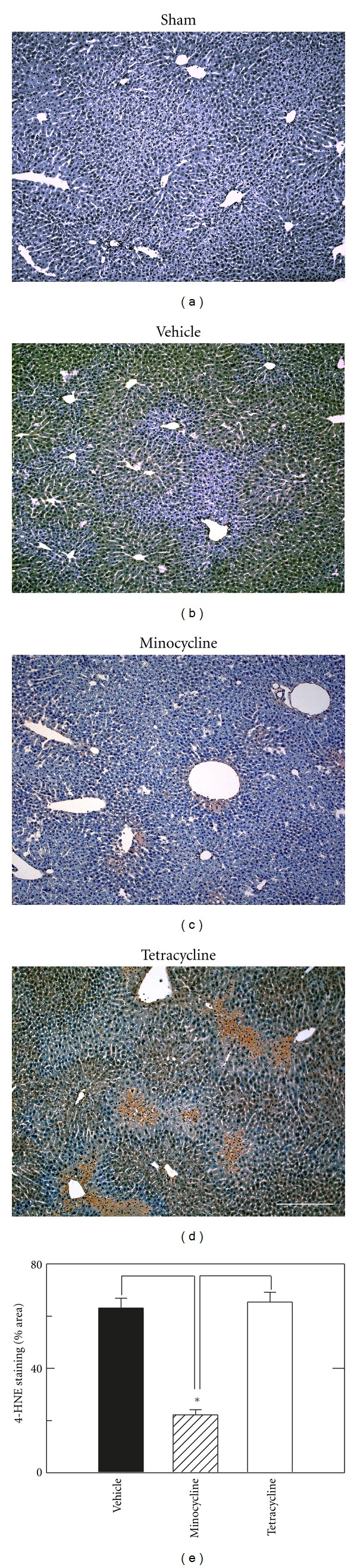
Minocycline decreases oxidative stress after hemorrhage and resuscitation. H/R was performed, as described in Figure 1, and immunohistochemical staining was performed for 4-HNE adducts at 6 h after sham operation (a) or resuscitation with vehicle, minocycline, or tetracycline (b–d). In (e), HNE staining as percent area in liver sections was averaged from 5 livers per group. HNE in sham-operated livers was virtually zero and not plotted. Individual group size was 5. Bar is 50 *μ*m. *P < 0.05 versus vehicle.

We used 4-HNE immunohistochemistry to evaluate oxidative stress in livers 6 h after hemorrhage and resuscitation. HNE is an aldehyde product of lipid peroxidation that forms covalent adducts with proteins that are recognized by anti-HNE antibodies. After sham operation, the brown reaction product of HNE immunohistochemistry was virtually undetectable (Figure 5(a)). By contrast at 6 h after resuscitation with vehicle or tetracycline, wide confluent areas of HNE immunoreactivity developed in pericentral and midzonal areas with relative sparing the periportal regions (Figures 5(b) and 5(d)). However, after H/R with minocycline, HNE immunoreactivity was decreased about 70% compared to vehicle and tetracycline treatments. HNE staining with minocycline was confined mostly to pericentral regions. (*P* < 0.05 compared to vehicle and tetracycline, Figures 5(c) and 5(e)).

### 3.4. Mitochondrial Dysfunction In Vivo after Hemorrhage and Resuscitation: Protection by Minocycline

**Figure 6:**
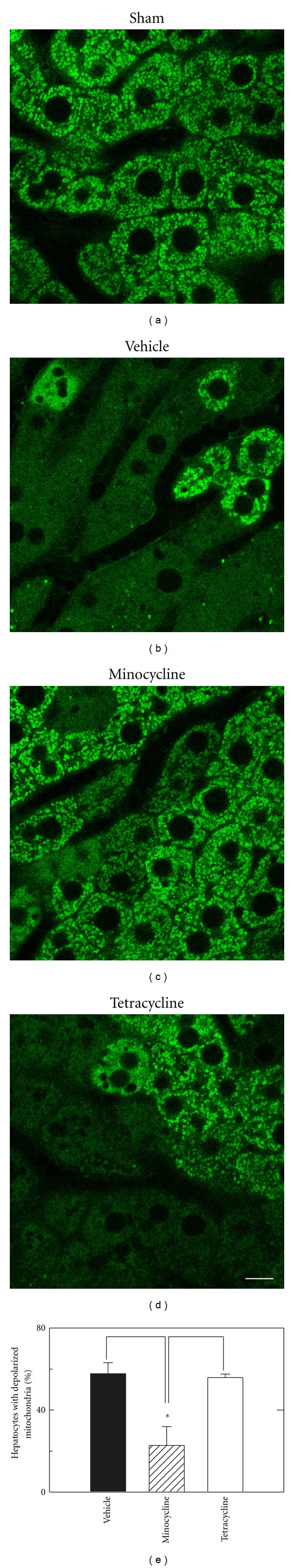
Minocycline decreases mitochondrial depolarization after hemorrhage and resuscitation. H/R was performed, as described in Figure 1, and intravital multiphoton microscopy of rhodamine 123 fluorescence was performed 4 h after sham operation (a) or resuscitation with vehicle, minocycline, or tetracycline (b–d), as described in materials and methods. Punctate staining of rhodamine 123 denoted polarization of individual mitochondria, whereas dim diffuse cellular staining indicated mitochondrial depolarization. In (e), the average percentage of hepatocytes with depolarized mitochondria is plotted for each H/R treatment group. Mitochondrial depolarization in sham-operated livers was virtually zero and not plotted. Size of individual groups was 5. Bar is 30 *μ*m. *P < 0.05 versus vehicle.

At 4 h after sham operation, intravital multiphoton microscopy revealed bright fluorescence of rhodamine 123 in virtually all hepatocytes whose punctate pattern signified polarization of individual mitochondria and normal mitochondrial function (Figure 6(a)). Cytosolic and nuclear areas had little fluorescence. By contrast at 4 h after H/R with vehicle treatment, rhodamine 123 staining became diffuse and dim in many hepatocytes (Figure 6(b)), which indicated mitochondrial depolarization and dysfunction. Similar to the necrosis and HNE immunoreactivity that became present at 6 h after H/R (see Figures 2 and 5), mitochondrial depolarization after 4 h had a predominantly pericentral and midzonal distribution (data not shown). After H/R with minocycline, fewer hepatocytes contained depolarized mitochondria (Figure 6(c)), whereas mitochondrial depolarization after tetracycline treatment was indistinguishable from vehicle-treated liver after H/R (Figure 6(d)). At 4 h postoperatively, livers were scored and counted for rhodamine 123 staining (Figure 6(e)). In sham-operated mice, 0.05 ± 0.002 hepatocytes/HPF contained depolarized mitochondria. After H/R with vehicle treatment, 12.2 ± 0.9 hepatocytes/HPF contained depolarized mitochondria, which corresponded to depolarization of 57.8 ± 5.2% of hepatocytes. After H/R with minocycline treatment, hepatocytes with depolarized mitochondria decreased to 5.4 ± 0.7 hepatocytes/HPF (*P* < 0.05 versus vehicle and tetracycline). By contrast, after H/R with tetracycline, 12.7 ± 0.9 hepatocytes/HPF contained depolarized mitochondria, which was not different from vehicle treatment (Figure 6(e)). 

## 4. Discussion

Hemorrhage is a risk of trauma and major surgery, particularly gastrointestinal and hepatobiliary surgery, and tissue damage after hemorrhage and resuscitation is a variant of ischemia/reperfusion injury. Despite advances in medical and surgical treatment, the golden hour for resuscitation remains a time limit and barrier to effective treatment of hemorrhagic shock. Moreover, the liver is among the most frequently affected organs after hemorrhage-induced hypotension in humans [5]. Here in a mouse model of H/R, we show that minocycline substantially decreases hepatic injury after resuscitation following 3 h of profound hemorrhagic hypotension. Specifically after 3 h of hemorrhage followed by resuscitation with shed blood and then lactated Ringers solution, hepatic necrosis, apoptosis, and enzyme release decreased by 50% or more after minocycline treatment (Figures 1–4). Minocycline also improved mitochondrial function as assessed by intravital multiphoton imaging of the fluorescence of the mitochondrial membrane potential-indicating fluorophore, rhodamine 123 (Figure 6). Notably, minocycline protected even when used after blood resuscitation as a component of Ringer's solution.

Previous studies show cytoprotection by minocycline in a variety of settings, including rat liver transplantation, ischemic renal injury, and various injuries to the central nervous system [7–12]. In our model of mouse H/R, minocycline protected even when used late during resuscitation after the initial blood resuscitation. Hepatic necrosis assessed by ALT and histology decreased by half at 6 h after H/R with minocycline treatment, and apoptosis assessed by TUNEL and caspase-3 activity also decreased by more than half (Figures 1–4). Necrosis represents the predominant mode of cell death in the setting of hepatic I/R with apoptosis contributing to a lesser extent [21, 22]. However, both modes of cell death, namely, apoptosis progressing to necrosis, can occur through a common mitochondrial pathway involving the MPT, a phenomenon of necrapoptosis [23–25].

After orthotopic rat liver transplantation, minocycline cytoprotection against hepatic necrosis, apoptosis, and enzyme release is virtually identical to the cytoprotection of *N*-methyl-4-isoleucine cyclosporin (NIM811), a specific inhibitor of the MPT [7]. Minocycline also inhibits calcium-induced MPT onset in isolated mitochondria. Unlike NIM811 which inhibits the MPT pore component, cyclophilin D, minocycline prevents MPT onset by blocking electrogenic calcium uptake by the mitochondrial calcium uniporter. Since H/R caused mitochondrial depolarization that was virtually identical to mitochondrial depolarization after liver transplantation, and since minocycline protected against this depolarization (Figure 6), it is likely the minocycline protects against hepatic injury after H/R by blocking MPT onset. Importantly, minocycline-sensitive mitochondrial depolarization signifying the MPT preceded necrotic cell death and thus was not a consequence of cell death, since after 4 h few cells labeled with propidium iodide, a marker of nonviable cells, as described previously [7]. Tetracycline, which did not decrease hepatic necrosis and ALT release after H/R, did not prevent mitochondrial depolarization after H/R (Figure 6). Because minocycline protected against mitochondrial depolarization, necrosis, and apoptosis, liver damage after H/R would appear to be largely a necroapoptotic phenomenon [26].

In storage/reperfusion injury during liver transplantation and in isolated mitochondria, tetracycline does not protect against hepatic damage, mitochondrial depolarization, and onset of the MPT [7]. Similarly in the present work, tetracycline did not protect against hepatic necrosis, enzyme release, and mitochondrial depolarization after H/R (Figures 1, 2 and 6). By contrast, tetracycline protected similarly to minocycline against apoptosis, as assessed by TUNEL and caspase 3 (Figures 3 and 4). This finding suggests different protective actions—one unique to minocycline and another shared by both tetracycline and minocycline. One shared action is that minocycline and tetracycline are both calcium chelators [27, 28], although only minocycline blocks mitochondrial calcium uptake [7]. Thus, suppression of apoptosis by tetracycline and minocycline might be due to calcium chelation. Alternatively in necrapoptosis, apoptosis progresses to necrosis with increasing severity of an inducing stress. Consequently, protective strategies may revert necrosis to apoptosis, such that protected necrotic areas begin to show apoptosis. Accordingly, protection against apoptosis by an agent like minocycline may be offset in part by increased apoptosis in areas that otherwise would have become necrotic. Tetracycline, by contrast, did not decrease necrosis, and tetracycline may simply represent a much weaker protective agent than minocycline that protects partially against apoptosis but not at all against necrosis. Future studies will be needed to distinguish between these possibilities.

In I/R, oxidative stress after reperfusion promotes the MPT, and antioxidants are protective. After H/R in our mouse model, 4-HNE immunostaining increased substantially as an indicator of lipid peroxidation and oxidative stress (Figure 5). Minocycline decreased this 4-HNE staining after H/R. Since minocycline is not an antioxidant, decreased HNE staining by minocycline suggests that oxidative stress is occurring as a consequence of the MPT and cell death. However, much HNE staining occurred in regions that had not yet become necrotic, and this oxidative stress might nonetheless be contributing to the progression of injury.

Endotoxin acting through lipopolysaccharide-binding protein contributes to H/R injury to liver [15]. As an antibiotic, minocycline might alter intestinal flora and hence endotoxemia after H/R. However, tetracycline is also a broad spectrum antibiotic, and tetracycline did not protect after H/R. The danger of bacterial infection necessitates prophylactic use of antibiotics, such as broad spectrum cephalosporins, after multiple trauma and in advance of major surgery [29–31]. Since minocycline is a broad spectrum antibiotic with an excellent safety record, one-time treatment of hemorrhagic shock patients with minocycline would be consistent with current clinical practice and has the additional benefit of decreasing injury from H/R and the subsequent development of MODS. Future studies will be needed to determine what benefit, if any, minocycline might have in a clinical setting of hemorrhagic shock and resuscitation.

## List of Abbreviations

4HNE: 4-Hydroxynonenal
ALT: Alanine aminotransferase
ATP: Adenosine triphosphate
H/R: Hemorrhage and resuscitation
H&E: Hematoxylin and eosin
HEPES: 4-(2-Hydroxyethyl)-1-piperazineethanesulfonic acid
HPF: High power field
MODS: Multiple organ dysfunction syndrome
MPT: Mitochondrial permeability transition
NIM811: *N*-Methyl-4-isoleucine cyclosporin
PT: Permeability transition
SIRS: Systemic inflammatory response syndrome
TUNEL: Terminal deoxynucleotidyl transferase-mediated dUTP nick-end labeling.
